# Weight loss, visit-to-visit body weight variability and cognitive function in older individuals

**DOI:** 10.1093/ageing/afac312

**Published:** 2023-01-09

**Authors:** Michelle H Zonneveld, Raymond Noordam, Behnam Sabayan, David J Stott, Simon P Mooijaart, Gerard J Blauw, J Wouter Jukema, Naveed Sattar, Stella Trompet

**Affiliations:** Department of Cardiology, Leiden University Medical Centre, Leiden, The Netherlands; Department of Internal Medicine, Section of Gerontology and Geriatrics, Leiden University Medical Center, Leiden, The Netherlands; Department of Internal Medicine, Section of Gerontology and Geriatrics, Leiden University Medical Center, Leiden, The Netherlands; HealthPartners Institute, Neuroscience Center, Bloomington, MN, USA and University of Minnesota, School of Public Health, Division of Epidemiology and Community Health; Institute of Cardiovascular and Medical Sciences, BHF Glasgow Cardiovascular Research Centre, University of Glasgow, Glasgow, UK; Department of Internal Medicine, Section of Gerontology and Geriatrics, Leiden University Medical Center, Leiden, The Netherlands; Department of Internal Medicine, Section of Gerontology and Geriatrics, Leiden University Medical Center, Leiden, The Netherlands; Department of Cardiology, Leiden University Medical Centre, Leiden, The Netherlands; Netherlands Heart Institute, Utrecht, The Netherlands; Institute of Cardiovascular and Medical Sciences, BHF Glasgow Cardiovascular Research Centre, University of Glasgow, Glasgow, UK; Department of Internal Medicine, Section of Gerontology and Geriatrics, Leiden University Medical Center, Leiden, The Netherlands

**Keywords:** weight loss, cognitive function, cognition, dementia, older adults, weight change, cognitive decline, older people

## Abstract

**Objective:**

to investigate the association between variability and loss of body weight with subsequent cognitive performance and activities of daily living in older individuals.

**Design:**

cross-sectional cohort study.

**Setting:**

PROspective Study of Pravastatin in the Elderly at Risk, multicentre trial with participants from Scotland, Ireland and the Netherlands.

**Subjects:**

4,309 participants without severe cognitive dysfunction (mean age 75.1 years, standard deviation (SD) = 3.3), at higher risk for cardiovascular disease (CVD).

**Methods:**

body weight was measured every 3 months for 2.5 years. Weight loss was defined as an average slope across all weight measurements and as ≥5% decrease in baseline body weight during follow-up. Visit-to-visit variability was defined as the SD of weight measurements (kg) between visits. Four tests of cognitive function were examined: Stroop test, letter-digit coding test (LDCT), immediate and delayed picture-word learning tests. Two measures of daily living activities: Barthel Index (BI) and instrumental activities of daily living (IADL). All tests were examined at month 30.

**Results:**

both larger body weight variability and loss of ≥5% of baseline weight were independently associated with worse scores on all cognitive tests, but minimally with BI and IADL. Compared with participants with stable weight, participants with significant weight loss performed 5.83 seconds (95% CI 3.74; 7.92) slower on the Stroop test, coded 1.72 digits less (95% CI −2.21; −1.13) on the LDCT and remembered 0.71 pictures less (95% CI -0.93; −0.48) on the delayed picture-word learning test.

**Conclusion:**

in older people at higher risk for CVD, weight loss and variability are independent risk-factors for worse cognitive function.

## Key Points

- In this study, we investigated whether loss of weight and variability in weight are associated with worsened cognitive function.
- Other markers of unstable homeostasis, such as higher variability in lipoprotein cholesterol and systolic blood pressure, have also been associated with worsened cognitive function.
- We found that higher visit-to-visit variability and overall weight loss were independently associated with a worse performance in three cognitive domains: memory, processing speed and selective attention.
- Our study suggests that variation in weight at older age may indicate increased cognitive vulnerability.

## Introduction

The process of ageing is accompanied by fluctuations in homeostatic processes, resulting in intrinsic intraindividual variability in physiological parameters. For example, variability in weight, including both gaining and losing weight, is associated with significant increases in mortality [1, 2]. Several observational studies have demonstrated that ‘unintentional’ weight loss in older adults is related to increased frailty and functional decline [2–5]. The origin of unintentional weight loss in older adults is often linked to the manifestation of malignancies, but numerous social, behavioural and health factors may also be instrumental [6, 7]. Over 27% of frail older individuals above the age of 65 years experience unintentional weight loss [8], where a specific cause cannot be found in as many as 25% of cases [2].

Recent evidence indicates that unintentional weight loss in older individuals associates with brain atrophy and cognitive impairment, which are known to associate with Alzheimer’s disease [3, 6, 9]. Other markers of unstable homeostasis and intraindividual variability, including variability in systolic blood pressure and low-density lipoprotein cholesterol, have also been associated with worsened cognitive function [10, 11]. We hypothesised that larger variation in body weight, as well as loss of weight, is independently associated with lower cognitive performance, independent of baseline body mass index (BMI) and traditional risk factors. Therefore, in the present study, we investigated the association of 2.5-year body weight loss and variation in body weight with subsequent cognitive performance and activities of daily living in a cohort of older individuals at increased risk of cardiovascular disease (CVD) but without severe cognitive dysfunction at baseline.

## Methods

### Study design and participants

The data employed in the present study originates from the PROspective Study of Pravastatin in the Elderly at Risk (PROSPER). In all, 5,804 men and women aged 70–82 years from three countries (the Netherlands, Scotland and Ireland) were enrolled between December 1997 and May 1999 in a prospective, multicentre randomised trial in order to assess the safety and efficacy of pravastatin in reducing the risk of major vascular events. Participants were eligible for enrolment if they had pre-existing vascular disease or increased risk because of smoking, hypertension or diabetes.

During recruitment, the following exclusion criteria were applied: cognitive impairment (Mini-Mental Score Examination score < 24); history of malignancy within the past 5 years except localised basal cell carcinoma; recent stroke, transient ischemic attack (TIA), myocardial infarction, surgery or amputation for vascular disease ≤6 months before study entry. More details regarding exclusion criteria of PROSPER have been described elsewhere [12, 13]. The PROSPER study was approved by the Medical Ethics Committees of the three collaborating centres and complied with the Declaration of Helsinki. All participants gave written informed consent.

In the present study, the following inclusion criteria were applied: ≥1 out of the four cognitive tests scores at month 30 of follow-up; ≥2 weight measurements recorded between baseline and month 30 of follow-up with a maximum of 11 repetitive measurements.

### Data collection

#### Exposure variables

Participants in the PROSPER study were reviewed every 3 months and body weight was measured at each visit, resulting in a maximum of 11 repetitive weight measurements.

From the weight collected at baseline and follow-up visits, we computed two determinants: weight loss and weight variability. Visit-to-visit weight variability was calculated by means of the intraindividual standard deviation (SD) over each individual’s measurements between baseline and every 3 months up to 30 months of follow-up. Weight loss was defined using two methods: first by calculating an average slope across all weight measurements, followed by calculating a delta incorporating only the first and last weight measurements during follow-up. The average slope incorporating all weight assessments during follow-up was computed using a linear mixed model capable of handling missing data during the visits. By fitting a linear-mixed model over the various measurements during follow-up, an average slope of weight change was calculated per participant. Then, the delta of weight change from baseline to month 30 was calculated and defined according to the following classification: weight loss as ≥5% of body weight decreased from baseline to month 30; weight gain as ≥5% of weight increased; and stability if within <5% weight variation between baseline and month 30 of follow-up [2, 6]. Although no universally accepted definition of clinically relevant weight loss exists, previous observational studies have employed this definition [2, 6].

#### Cognitive function measurements

Serving as our outcome measurements, cognitive function was assessed face-to-face using four neuropsychological performance tests. More details regarding the tests are described in detail elsewhere [14]. The Stroop colour-word inference test (selective attention) and the letter-digit coding test (LDCT) (processing speed) were used to measure executive functioning [14]. The outcome parameter for the Stroop test was the total number of seconds to complete the third Stroop card. The outcome variable for the LDCT was the total number of correct entries in 60 s. Visual episodic memory was assessed with the 15-Picture Learning test (PLT) testing immediate and delayed recall. The main outcome was the accumulated number of recalled pictures over the three learning trials and the number of pictures recalled after 20 min. Functional status was assessed using the Barthel Index (BI) and instrumental activities of daily living (IADL). BI assesses self-care activities of daily living using 10 items (such as dressing) where a higher score indicates higher independence, with a maximum of 20 points [15]. Similarly, IADL measures activities of daily living using seven items with a maximum of 14 points, but in addition includes the interaction with the social and physical environment [16]. Likewise, here a higher score also higher functional capacity and independence. All outcomes were assessed at month 30 to maximise the availability of outcomes after the measurement of weight variability and weight change.

#### Covariates

Covariates were obtained from an extensive medical history using routine care data during a 10-week screening period. The participant’s general practitioner reported on the history of various clinical diseases, including CVD, diabetes mellitus, TIA, stroke and myocardial infarction. The following were evaluated using a medical inventory: number of medications used, use of diuretics and antidepressants, years of education, smoking status, alcohol intake. Systolic and diastolic blood pressure were measured every 3 months [10]. Data on diseases developed during follow-up were ascertained using hospital records and general practitioners’ records, and included: diabetes; non-fatal cardiovascular and coronary events including myocardial infarction, stroke and TIA; hospitalisation because of heart failure; serious non-fatal cancer [13]. These endpoints were classified by an independent committee. Data on these baseline covariates were complete for all participants.

#### Statistical analyses

Baseline characteristics of the study participants are reported as mean (SD) or median (interquartile range (IQR)) for continuous variables and number (percentage) for categorical variables. To investigate whether slope and variability were two independent phenotypes, we assessed the correlation with a Pearson’s correlation coefficient. We repeated the analyses with both slope of weight change and weight variability in the same multivariable-adjusted regression model to study whether these phenotypes have independent effects.

The associations of visit-to-visit variability in total weight (in SD), slope of weight change (in kg) and weight loss (in categories) with measures of cognitive function at month 30 were assessed using multivariable-adjusted linear regression models. Weight variability and slope of weight change were analysed as continuous variables and in equally sized thirds, where the lower third of weight variability and the middle third of the slope of weight change were defined as reference categories. For reasons of clinical interpretation, we presented the results on slope of weight change as the difference in cognitive function at month 30 per extra 0.1 kg/month weight loss.

The linear regression analyses were adjusted for covariates based on their biological plausibility as potential confounders. Therefore, in the minimally adjusted model, we included sex, age, country as a three-level variable, mean weight during follow-up, height at baseline and years of education. The fully adjusted model additionally included smoking status, alcohol intake, number of medications used, use of diuretics and antidepressants, and history of CVD, diabetes and myocardial infarction.

Data are reported as the mean multivariable-adjusted difference in outcome between the second and third thirds of weight change in comparison with the reference group, with accompanying 95% confidence interval. For example, ‘weight lost’ and ‘weight gained’ are compared with the reference group ‘stable weight’. All analyses were performed using SPSS Windows version 26 (IBM Corp., 2019). Data from the PROSPER study is not publicly available.

#### Sensitivity analyses

In addition to these overall analyses, we performed sensitivity analyses to investigate the robustness of the associations considering different subgroups of the population. First, we performed separate analyses for placebo and pravastatin treatment groups. Next, it has been shown that high blood pressure variability was associated with worse cognitive function [10]. As variability in weight and blood pressure may have a common cause, we additionally adjusted our models for systolic blood pressure variability and mean systolic blood pressure from baseline to month 30. This allows for the separation of effects of weight variability from those originating from blood pressure variability. Systolic blood pressure variability was defined as the intraindividual SD from baseline to month 30, where blood pressure was measured every 3 months, as previously done [10, 17]. Furthermore, we included both weight loss (slope) and visit-to-visit body weight variables in the same multivariable-adjusted linear regression model to test independence of the two phenotypes. Last, we performed analyses excluding individuals who developed any of the following diseases during follow-up, to ensure weight loss did not follow as a result: incident diabetes, non-fatal cancer, non-fatal stroke or TIA, hospitalisation because of heart failure and non-fatal coronary or cardiovascular events.

## Results

After excluding participants with <2 measurements (*n* =  225) and participants without cognitive test scores at month 30 (*n* = 1,270), 4,309 participants were eligible for inclusion (Table 1). The mean age was 75.1 years (SD = 3.3) and more than half of the study population was female (*n* = 2,222, 51.6%). Large majority of participants had a history of hypertension (*n* = 2,706, 62.8%). The mean weight during follow-up was 72.9 kg (SD = 13.3) and participants had a median weight loss of 0.01 kg per month (IQR −0.07; 0.06). Baseline weight characteristics per third of weight loss and weight variability are reported in Supplementary Table 1.

**Table 1:** Demographics and clinical characteristics of study population

Sociodemographics		All (*n* = 4,309)
Age, year, mean (SD)		75.1 (3.3)
Female, *n* (%)		2,222 (51.6)
Age left school, year, mean (SD)		15.2 (2.1)
Current smoker, *n* (%)		1,079 (25.0)
Alcohol intake, unit intake per week, mean (SD)		5.30 (9.2)
**Cardiovascular risk factors**		
History of CVD, *n* (%)		1878 (43.6)
History of hypertension, *n* (%)		2,706 (62.8)
History of stroke or TIA, *n* (%)		465 (10.8)
History of myocardial infarction, *n* (%)		560 (13.0)
Diabetes mellitus, *n* (%)		446 (10.4)
Serum cholesterol, mmol/L, mean (SD)		5.68 (0.9)
Weight during follow-up, kg, mean (SD)		72.9 (13.3)
Weight change, kg/month, mean (SD)		−0.01 (0.1)
Lost more than 5% of baseline body weight during follow-up, *n* (%)		802 (18.6)
Gained more than 5% of baseline body weight during follow-up, *n* (%)		580 (13.5)
Number of weight measurements, median (IQR)		10 (10; 10)
BMI, kg/m^2^, mean (SD)		26.9 (4.1)
Pravastatin treatment, *n* (%)		2,146 (49.8)
Number of medications, median (IQR)		3 (2; 5)
Use of diuretics, *n* (%)		1804 (41.9)
**Cognitive function at month 30 of follow-up**		
Stroop test, s, mean (SD)^a^		64.5 (26.1)
LDCT, digits coded, mean (SD)^b^		22.9 (7.8)
PLTi, pictures remembered, mean (SD)^c^		9.5 (2.0)
PLTd, pictures remembered, mean (SD)^c^		10.2 (2.9)
Barthel, index, mean (SD)^d^		19.7 (0.9)
IADL, points, mean (SD)^d^		13.5 (1.3)

*Notes*: PLTi, picture-word learning test immediate; PLTd, picture-word learning test delayed. ^a^Performed by 4,122 participants; ^b^performed by 4,264 participants; ^c^performed by 4,309 participants; ^d^performed by 4,428 participants.

The correlation between continuous weight variability and the continuous slope of weight change was negligible (Pearson’s *r* = 0.22).

### Association between weight loss and cognitive function

Table 2 displays the association of the slope of weight change and cognitive function. After full adjustments, in comparison to the middle third, the lower third of the slope of weight change was associated with a worse performance in all cognitive and functional domains except on the BI (−0.04 points, 95% CI −0.10; 0.03). To illustrate, at month 30 of follow-up, the lower third coded 1.42 (95% CI −1.98; −0.86) digits less on the LDCT and performed 4.39 s (95% CI 2.42; 6.37) slower on the Stroop test. On the other hand, in comparison to the middle third, the upper third of the slope of weight change was not significantly associated with cognitive performance. Continuously, the slope of weight change was also associated with worse cognitive function on all tests. Per 0.10 kg/month additional average weight loss, the score on the Stroop test was 1.82 s (95% CI 1.13; 2.49) slower, 0.70 less (95% CI −0.90; −0.51) digits were coded on the LDCT and 0.21 less (95% CI −0.29; −0.14) pictures were remembered on the delayed PLT.

**Table 2:** Associations of weight change (slope) during follow-up time and cognitive function at month 30 of follow-up

		Weight change (slope)			
		Low third	Middle third	Upper third	Continuous^c^
		(*N*_max_ = 1,436)	(*N*_max_ = 1,436)	(*N*_max_ = 1,437)	All (*N*_max_ = 4,309)
Cognitive test		Beta (95% CI)	Beta (95% CI)	Beta (95% CI)	Beta (95% CI)
**Minimally adjusted** ^**a**^					
Stroop, s		**4.75 (2.84; 6.67)**	Ref	0.34 (−1.52; 2.19)	**1.91 (1.25; 2.57)**
LDCT, digits coded		**−1.52 (−2.07; −0.97)**	Ref	0.32 (−0.21; 0.86)	**−0.69 (−0.88; −0.50)**
PLTi, pictures remembered		**−0.37 (−0.52; −0.23)**	Ref	**0.01 (−0.13; 0.15)**	**−0.15 (−0.20; −0.10)**
PLTd, pictures remembered		**−0.56 (−0.77; −0.36)**	Ref	−0.06 (−0.26; 0.14)	**−0.21 (−0.28; −0.14)**
Barthel, index		−0.04 (−0.10; 0.03)	Ref	0.00 (−0.06; 0.07)	−0.02 (−0.04; 0.00)
IADL, points		**−0.11 (−0.20; −0.02)**	Ref	−0.07 (−0.16; 0.02)	−0.04 (−0.07; 0.01)
**Fully adjusted** ^**b**^					
Stroop, s		**4.39 (2.42; 6.37)**	Ref	0.15 (−1.78; 2.06)	**1.82 (1.13; 2.49)**
LDCT, digits coded		**−1.42 (−1.98; −0.86)**	Ref	0.43 (−0.12; 0.97)	**−0.70 (−0.90; −0.51)**
PLTi, pictures remembered		**−0.37 (−0.52; −0.22)**	Ref	0.02 (−0.12; 0.17)	**−0.15 (−0.20; −0.10)**
PLTd, pictures remembered		**−0.52 (−0.73; −0.31)**	Ref	−0.01 (−0.22; 0.20)	**−0.21 (−0.29; −0.14)**
Barthel, index		−0.04 (−0.10; 0.03)	Ref	0.00 (−0.06; 0.07)	−0.02 (−0.05; 0.00)
IADL, points		**−0.11 (−0.21; −0.02)**	Ref	−0.07 (−0.16; 0.02)	**−0.04 (−0.07; −0.01)**

*Notes*: PLTi, picture-word learning test immediate; PLTd, picture-word learning test delayed. ^a^Sex, age, country, mean weight during follow-up, height, education. ^b^Sex, age, country, education level, height, mean weight during follow-up time, history of cardiovascular disease, history of diabetes, history of myocardial infarct, smoking, alcohol intake, number of medications, use of diuretics, use of antidepressants. ^c^Presented results can be interpreted as the difference is cognitive performance at month 30 per 0.1 kg/month additional weight loss. Stroop test was performed by 4,122 participants, LDCT was performed by 4,264 participants and the picture-word Learning immediate and delayed tests were performed by 4,309 participants. Items written in bold indicate that the 95% confidence interval does not contain 0.

**Figure 1:**
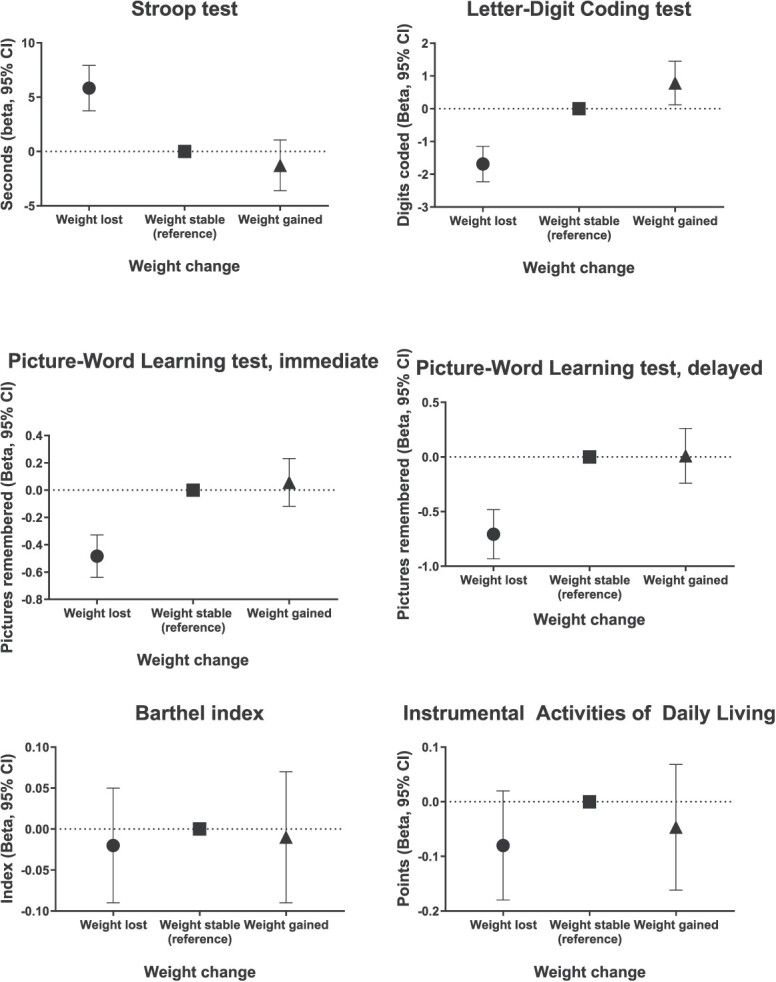
Fully adjusted associations of weight change during follow-up time and cognitive function at month 30. Weight loss was defined as ≥5% of body weight decreased from baseline to month 30, weight gain as ≥5% of weight increased and stability if within <5% weight variation between baseline and month 30 of follow-up.

Loss of ≥5% of weight during follow-up was associated with worse performance on all domains, but did not show association with BI and IADL (Table 3 and Figure 1). After full adjustments, in comparison to maintaining stable weight during follow-up, participants who lost ≥5% of baseline body weight performed 5.83 s (95% CI 3.74; 7.92) slower on the Stroop test. Furthermore, weight loss was also associated with a worse performance on the LDCT (Beta −1.72 digits coded, 95% CI −2.21; −1.13) and the PLT, both immediate (beta −0.48 pictures remembered, 95% CI −0.64; −0.33) and delayed (beta −0.71 pictures remembered, 95% CI −0.93; −0.48). In comparison to individuals who maintained stable weight, we did not find evidence of a significant association between weight gain during follow-up and cognitive function.

**Table 3:** Associations of weight change during follow-up time and cognitive function at month 30 of follow-up

		Weight change		
		Weight lost	Weight stable	Weight gained
		(*N*_max_ = 802)	(*N*_max_ = 2,927)	(*N*_max_ = 644)
Cognitive test		Beta (95% CI)	Beta (95% CI)	Beta (95% CI)
**Minimally adjusted** ^**a**^				
Stroop, s		**6.34 (4.31; 8.37)**	Ref	−0.74 (−3.02; 1.54)
LDCT, digits coded		**−1.85 (−2.43; −1.27)**	Ref	0.52 (−0.14; 1.17)
PLTi, pictures remembered		**−0.48 (−0.64; −0.33)**	Ref	0.02 (−0.15; 0.20)
PLTd, pictures remembered		**−0.74 (−0.96; −0.52)**	Ref	−0.08 (−0.32; 0.17)
Barthel, index		−0.02 (−0.09; 0.04)	Ref	−0.02 (−0.09; 0.06)
IADL, points		−0.09 (−0.19; 0.01)	Ref	−0.07 (−0.18; 0.04)
**Fully adjusted** ^**b**^				
Stroop, s		**5.83 (3.74; 7.92)**	Ref	−1.28 (−3.62; 1.06)
LDCT, digits coded		**−1.72 (−2.21; −1.13)**	Ref	**0.78 (0.12; 1.45)**
PLTi, pictures remembered		**−0.48 (−0.64; −0.33)**	Ref	0.06 (−0.12; 0.23)
PLTd, pictures remembered		**−0.71 (−0.93; −0.48)**	Ref	0.01 (−0.24; 0.26)
Barthel, index		−0.02 (−0.09; 0.05)	Ref	−0.01 (−0.09; 0.07)
IADL, points		−0.08 (−0.18; 0.02)	Ref	−0.05 (−0.16; 0.07)

*Notes*: PLTi, picture-word learning test immediate; PLTd, picture-word learning test delayed. ^a^Sex, age, country, mean weight during follow-up, height, education. ^b^Sex, age, country, education level, height, mean weight during follow-up time, history of cardiovascular disease, history of diabetes, history of myocardial infarct, smoking, alcohol intake, number of medications, use of diuretics, use of antidepressants. Weight loss was defined as ≥5% of body weight decreased from baseline to month 30, weight gain as ≥5% of weight increased and stability if within <5% weight variation between baseline and month 30 of follow-up. Stroop test was performed by 4,122 participants, LDCT was performed by 4,264 participants and the picture-word learning immediate and delayed tests were performed by 4,309 participants. Items written in bold indicate that the 95% confidence interval does not contain 0.

### Association between visit-to-visit body weight variability and cognitive function

The associations of visit-to-visit weight variability and cognitive function are presented in Table 4. After full adjustments, both middle and upper thirds of weight variability, in comparison to the lower third, performed worse on all tests. For example, the middle third (moderate variability) of weight variability was associated with 1.93 s (95% CI 0.01; 3.86) slower performance on the Stroop test, whereas the upper third (high variability) was associated with 4.52 s (95% CI 2.52; 6.51) slower performance, in comparison to the lower third (low variability). Likewise, higher weight variability as a continuous variable was associated with worse cognitive function on all domains. Here, a 1-SD higher weight variability was associated with 1.46 s (95% CI 0.82; 2.09) slower performance on the Stroop test. There were no associations with functional capacity.

**Table 4:** Associations of weight variability (SD) and cognitive function at month 30 of follow-up

		Weight variability, SD			
		Low third	Middle third	Upper third	Continuous
		(*N*_max_ = 1,436)	(*N*_max_ = 1,436)	(*N*_max_ = 1,437)	All (*N*_max_ = 4,309)
Cognitive test		Beta (95% CI)	Beta (95% CI)	Beta (95% CI)	Beta (95% CI)
**Minimally adjusted** ^**a**^					
Stroop, s		Ref	**2.30 (0.42; 4.18)**	**6.01 (4.07; 7.95)**	**1.98 (1.35; 2.60)**
LDCT, digits coded		Ref	**−1.42 (−1.96; −0.88)**	**−2.29 (−2.85; −1.74)**	**−0.73 (−0.91; −0.55)**
PLTi, pictures remembered		Ref	**−0.21 (−0.35; −0.06)**	**−0.63 (−0.78; −0.49)**	**−0.19 (−0.24; −0.14)**
PLTd, pictures remembered		Ref	**−0.21 (−0.35; −0.06)**	**−0.96 (−1.16; −0.75)**	**−0.29 (−0.35; −0.22)**
Barthel, index		Ref	−0.04 (−0.10; 0.03)	**−0.09 (−0.15; −0.02)**	**−0.06 (−0.08; −0.04)**
IADL, points		Ref	−0.04 (−0.13; 0.05)	**−0.19 (−0.28; −0.10)**	**−0.10 (−0.13; −0.07)**
**Fully adjusted** ^**b**^					
Stroop, s		Ref	**2.25 (0.31; 4.18)**	**5.32 (0.31; 4.18)**	**1.72 (1.08; 2.35)**
LDCT, digits coded		Ref	**−1.36 (−1.92; −0.81)**	**−2.16 (−2.72; −1.59)**	**−0.67 (−0.86; −0.49)**
PLTi, pictures remembered		Ref	**−0.19 (−0.33; −0.04)**	**−0.61 (−0.76; −0.46)**	**−0.18 (−0.23; −0.13)**
PLTd, pictures remembered		Ref	−0.20 (−0.41; 0.01)	**−0.91 (−1.12; −0.69)**	**−0.27 (−0.33; −0.20)**
Barthel, index		Ref	−0.04 (−0.11; 0.02)	**−0.08 (−0.15; −0.02)**	**−0.05 (−0.08; −0.03)**
IADL, points		Ref	−0.05 (−0.14; 0.04)	**−0.17 (−0.27; −0.08)**	**−0.09 (−0.12; −0.06)**
**Fully adjusted with systolic blood pressure variability** ^**c**^					
Stroop, s		Ref	**1.93 (0.01; 3.86)**	**4.52 (2.52; 6.51)**	**1.46 (0.82; 2.09)**
LDCT, digits coded		Ref	**−1.28 (−1.83; −0.73)**	**−1.95 (−2.51; −1.39)**	**−0.61 (−0.79; −0.43)**
PLTi, pictures remembered		Ref	**−0.17 (−0.31; −0.03)**	**−0.58 (−0.73; −0.43)**	**−0.17 (−0.22; −0.12)**
PLTd, pictures remembered		Ref	−0.18 (−0.39; 0.02)	**−0.86 (−1.08; −0.65)**	**−0.25 (−0.32; −0.19)**
Barthel, index		Ref	−0.04 (−0.10; 0.03)	**−0.08 (−0.14; −0.01)**	**−0.05 (−0.07; −0.03)**
IADL, points		Ref	−0.04 (−0.14; 0.05)	**−0.17 (−0.25; −0.06)**	**−0.08 (−0.11; −0.05)**

*Notes*: PLTi, picture-word learning test immediate; PLTd, picture-word learning test delayed. ^a^Sex, age, country, mean weight during follow-up, height, education. ^b^Sex, age, country, education level, height, mean weight during follow-up time, history of cardiovascular disease, history of diabetes, history of myocardial infarct, smoking, alcohol intake, number of medications, use of diuretics, use of antidepressants. ^c^Sex, age, country, education level, height, mean weight during follow-up time, history of cardiovascular disease, history of diabetes, history of myocardial infarct, smoking, alcohol intake, number of medications, use of diuretics, use of antidepressants, systolic blood pressure variability, mean systolic blood pressure. Stroop test was performed by 4,122 participants, LDCT was performed by 4,264 participants and the picture-word learning immediate and delayed tests were performed by 4,309 participants. Items written in bold indicate that the 95% confidence interval does not contain 0.

### Sensitivity analyses

Adjusting the analyses between weight variability and cognitive function for systolic blood pressure variability did not materially change the results (Table 4). The results were essentially unchanged when stratifying for the treatment groups (Supplementary Tables 2–4). Repeating the analyses after excluding participants with incident disease-states during follow-up also did not materially change the associations (Supplementary Tables 5–7). The associations between continuous weight variability and continuous slope of weight change with cognitive function remained significant when including the two determinants in the same model (Supplementary Table 8).

## Discussion

In the present study, we investigated the association of 2.5-years variation in weight and loss of weight with subsequent cognitive performance in a cohort of older individuals at increased risk of CVD. Loss of weight and a higher weight variability were independently significantly associated with worse cognition. These findings were consistent in all tested cognitive domains and independent of incident disease-states, use of diuretics or antidepressants, cardiovascular risk-factors. This study found no associations with activities of daily living.

Major strengths of this study are its size with >4,300 older participants, use of multiple consecutive weight measurements and the ability to investigate various cognitive domains. Furthermore, participants were free of dementia at baseline because of the PROSPER exclusion criteria of MMSE scores < 24. A limitation is the lack of information regarding physical activity and intentional weight loss. At the onset of data collection, study participants received health counselling that may have led some participants to intentionally lose weight. Intentional loss of weight has been showed to result in improved cognitive function [18], whereas the present study could not corroborate this observation. Furthermore, reverse causation may also play a role as cognitive impairment can also lead to raised energy expenditure or changes in eating habits [2]. Lack of associations of weight loss or variability with BI and IADL may be because of the ceiling effects.

Current evidence on the association between cognitive decline and variation of weight mainly comes from studies that collected fewer weights measurements (≤3) than the present study (median of 11 measurements, IQR 11; 11) [1, 6, 19, 20]. Furthermore, these studies did not calculate an average slope of weight change during follow-up as done in the present study. In line with our results, these studies demonstrate that greater variation in weight is associated with higher risk of dementia. In the present study, we used a battery of cognitive tests to examine various domains as opposed to solely the diagnosis of dementia [1, 19–21], adding nuance to our findings. Consistent with our findings, these studies suggest that larger variation in weight may function as a marker of risk of early cognitive impairment.

On the other hand, some studies present mixed results. Improved cognitive function following intended weight loss has also been observed amongst older individuals [18, 22, 23]. However, these studies were designed to ‘intentionally’ induce weight loss in participants, whereas in the present study, it is believed that weight loss in the vast majority was ‘unintentional’ and therefore a consequence of other subclinical processes.

Incident disease-states such as cancer are often thought to be the underlying cause of weight loss [2]. In the present study, participants with a recent history of malignancies (<5 years) and cardiovascular events (<6 months) were excluded during recruitment of the original PROSPER trial, and we found that associations did not change after repeating the analyses excluding participants with incident disease-states during follow-up. It is therefore more likely that weight loss in the present study may have resulted from unstable homeostasis rather than because of major disease during the follow-up. We also demonstrated that higher weight variability was associated with worse cognitive function, independent of systolic blood pressure variability. This may suggest that variability in body weight and systolic blood pressure do not share a common cause, and that the current findings are different from what we have previously published [10, 11]. In addition, the two variability variables that are more likely to symbolise different biological pathways are involved in regulating homeostasis.

The biological mechanisms by which weight loss and most notably weight variability is associated with cognitive function are not fully understood. Weight loss can result as a downstream effect of normal ageing as metabolic needs may change [2]. In addition, polypharmacy can cause dentition and absorption issues, altered gastric signals, causing early satiation or loss of appetite. Reverse causality, where weight loss is an early manifestation of dementia [24], could also contribute to our findings. However, weight loss has been shown to precede symptoms of cognitive decline, implying that pathological processes of weight loss could contribute, perhaps indirectly, to cognitive decline [25]. Future long-term investigations are warranted to examine whether maintaining weight stability can effectively decrease the risk of cognitive decline.

In conclusion, we found that in older participants at increased risk for vascular disease, steeper decline in weight and a higher variability in body weight were strongly associated with lower cognitive function in multiple domains. These findings were independent of cardiovascular risk-factors, comorbidities, incident disease-states and independent of each other. Although we did not produce evidence favouring weight change to cause decreased cognitive function, it may represent an early manifestation or signal of cognitive decline.

## Supplementary Material

aa_21_1822_File002_afac312
