# Supplementary material for: Weight loss, visit-to-visit body weight variability and cognitive function in older individuals
Source: Age Ageing. 2023 Jan 9;52(1):afac312. doi: 10.1093/ageing/afac312 (PMC9990986; doi:10.1093/ageing/afac312)
Supplement: aa_21_1822_File002_afac312 [file aa_21_1822_file002_afac312.docx]

Weight Loss, Visit-To-Visit Body Weight Variability and Cognitive Function in Older Individuals

Contents of supplementary material

[**Table S1.** Baseline weight characteristics per third 2](#_Toc110267472)

[**Table S2.** Associations of weight variability (SD) and cognitive function at month 30 of follow-up. 3](#_Toc110267473)

[**Table S3.** Associations of weight change (slope) during follow-up time and cognitive function at month 30 of follow-up. 4](#_Toc110267474)

[**Table S4.** Associations of weight change during follow-up time and cognitive function at month 30 of follow-up. 5](#_Toc110267475)

[**Table S5.** Cross-sectional associations of weight variability (SD) and cognitive function at month 30 of follow-up – individuals with disease during follow-up excluded. 6](#_Toc110267476)

[**Table S6.** Cross-sectional associations of weight change (slope) during follow-up time and cognitive function at month 30 of follow-up - individuals with disease during follow-up excluded. 7](#_Toc110267477)

[**Table S7.** Cross-sectional associations of weight change during follow-up time and cognitive function at month 30 of follow-up - individuals with disease during follow-up excluded. 8](#_Toc110267478)

[**Table S8.** Continuous weight variability and continuous weight change (slope) in the same model. 9](#_Toc110267479)

# **Table S1.** Baseline weight characteristics per third

|  | | **Baseline weight** | **Obese (BMI>30)** | **Weight change^1^** | **Weight variability in SD^1^** |
| --- | --- | --- | --- | --- | --- |
|  | | **Mean (SD), kg** | **N (%)** | **Mean (SD), kg** | **Mean (SD)** |
| **Weight change (slope)** | |  |  |  |  |
|  | Low third | 74.22 (13.94) | 338 (24.20) | -4.71 (3.94) | 2.52 (1.40) |
|  | Middle third | 72.74 (12.42) | 275 (19.00) | -0.57 (1.95) | 1.50 (0.91) |
|  | Upper third | 74.61 (13.44) | 309 (19.50) | 3.09 (2.99) | 2.18 (1.21) |
| **Weight change^2^** | |  |  |  |  |
|  | Weight lost | 73.37 (13.99) | 208 (25.00) | -6.65 (3.83) | 2.85 (1.58) |
|  | Weight stable | 74.61 (12.97) | 630 (21.00) | -0.11 (0.41) | 1.73 (0.93) |
|  | Weight gained | 70.88 (13.49) | 84 (14.10) | 5.81 (2.68) | 2.64 (1.47) |
| **Weight variability** | |  |  |  |  |
|  | Low third | 70.55 (11.88) | 204 (14.40) | -0.11 (1.95) | 1.12 (0.25) |
|  | Middle third | 73.71 (12.63) | 282 (18.30) | -0.27 (3.17) | 1.78 (0.20) |
|  | Upper third | 77.24 (14.40) | 436 (29.60) | -1.27 (6.62) | 3.27 (1.50) |

**^1^**=Between baseline and month 30 of follow-up. **^2^**=Weight loss was defined as ≥5% of body weight decreased from baseline to month 30; weight gain as ≥5% of weight increased; and stability if within <5% weight variation between baseline and month 30 of follow-up.

# **Table S2.** Associations of weight variability (SD) and cognitive function at month 30 of follow-up.

|  | | **Weight variability, SD** | | | | | | |
| --- | --- | --- | --- | --- | --- | --- | --- | --- |
|  | | **Low third** | **Middle third** | **Middle third** | **Upper third** | **Upper third** | **Continuous** | **Continuous** |
|  | |  | **Placebo** | **Pravastatin** | **Placebo** | **Pravastatin** | **Placebo** | **Pravastatin** |
| **Cognitive test** | | **Beta (95% CI)** | **Beta (95% CI)** | **Beta (95% CI)** | **Beta (95% CI)** | **Beta (95% CI)** | **Beta (95% CI)** | **Beta (95% CI)** |
| **Minimally adjusted^1^** | |  |  |  |  |  |  |  |
|  | Stroop, seconds | Ref | **3.44 (0.71; 6.17)** | 1.18 (-1.41; 3.77) | **6.90 (4.12; 9.68)** | **5.11 (2.39; 7.82)** | **1.87 (0.97; 2.76)** | **2.07 (1.19; 2.95)** |
|  | LDCT, digits coded | Ref | **-1.55 (-2.31; -0.78)** | **-1.29 (-2.06; -0.52)** | **-2.10 (-2.88; -1.33)** | **-2.50 (-3.30; -1.69)** | **-0.61 (-0.86; -0.36)** | **-0.86 (-1.12; -0.60)** |
|  | PLTi, pictures remembered | Ref | -0.18 (-0.39; 0.02) | **-0.22 (-0.42; -0.03)** | **-0.61 (-0.82; -0.41)** | **-0.66 (-0.86; -0.45)** | **-0.16 (-0.22; -0.10)** | **-0.22 (-0.29; -0.16)** |
|  | PLTd, pictures remembered | Ref | **-0.29 (-0.57; -0.01)** | -0.14 (-0.42; 0.14) | **-1.09 (-1.38; -0.80)** | **-0.81 (-0.11; -0.52)** | **-0.26 (-0.36; -0.17)** | **-0.31 (-0.41; -0.22)** |
|  | Barthel, index | Ref | -0.02 (-0.10; 0.07) | -0.06 (-0.15; 0.04) | **-0.09 (-0.18; -0.01)** | -0.08 (-0.18; 0.04) | **-0.06 (-0.08; -0.03)** | **-0.06 (-0.09; -0.03)** |
|  | IADL, points | Ref | -0.02 (-0.15; 0.10) | -0.07 (-0.19; 0.06) | **-0.20 (-0.32; -0.07)** | **-0.18 (-0.32; -0.05)** | **-0.08 (-0.12; -0.04)** | **-0.12 (-0.16; -0.07)** |
| **Fully adjusted^2^** | |  |  |  |  |  |  |  |
|  | Stroop, seconds | Ref | **3.43 (0.61; 6.26)** | 1.13 (-1.53; 3.78) | **6.57 (3.70; 9.45)** | **4.03 (1.23; 6.83)** | **1.60 (0.68; 2.51)** | **1.82 (0.93; 2.71)** |
|  | LDCT, digits coded | Ref | **-1.54 (-2.32; -0.75)** | **-1.20 (-1.99; -0.42)** | **-2.09 (-2.89; -1.30)** | **-2.21 (-3.03; -1.39)** | **-0.58 (-0.83; -0.33)** | **-0.77 (-1.03; -0.51)** |
|  | PLTi, pictures remembered | Ref | -0.15 (-0.36; 0.06) | **-0.22 (-0.42; -0.02)** | **-0.60 (-0.81; -0.39)** | **-0.62 (-0.83; -0.40)** | **-0.15 (-0.22; -0.09)** | **-0.21 (-0.28; -0.14)** |
|  | PLTd, pictures remembered | Ref | -0.28 (-0.58; 0.02) | -0.13 (-0.42; 0.16) | **-1.08 (-1.38; -0.78)** | **-0.71 (-1.02; -0.41)** | **-0.25 (-0.35; -0.16)** | **-0.28 (-0.38; -0.18)** |
|  | Barthel, index | Ref | -0.01 (-0.10; 0.07) | -0.07 (-0.17; 0.03) | -0.09 (-0.18; 0.00) | -0.07 (-0.17; 0.04) | **-0.05 (-0.08; -0.03)** | **-0.05 (-0.09; -0.02)** |
|  | IADL, points | Ref | -0.02 (-0.15; 0.11) | -0.07 (-0.20; 0.06) | **-0.18 (-0.31; -0.05)** | -0.15 (-0.29; 0.00) | **-0.07 (-0.11; -0.03)** | **-0.11 (-0.15; -0.06)** |
| **Fully adjusted with systolic blood pressure variability^3^** | | | |  |  |  |  |  |
|  | Stroop, seconds | Ref | **2.94 (0.13; 5.75)** | 1.03 (-1.62; 3.68) | **5.65 (2.79; 8.52)** | **3.39 (0.60; 6.19)** | **1.30 (0.39; 2.21)** | **1.61 (0.72; 2.50)** |
|  | LDCT, digits coded | Ref | **-1.38 (-2.16; -0.60)** | **-1.20 (-1.98; -0.42)** | **-1.85 (-2.64; -1.06)** | **-2.05 (-2.86; -1.23)** | **-0.51 (-0.76; -0.25)** | **-0.71 (-0.98; -0.45)** |
|  | PLTi, pictures remembered | Ref | -0.12 (-0.33; 0.09) | **-0.22 (-0.42; -0.02)** | **-0.56 (-0.77; -0.35)** | **-0.58 (-0.80; 0.37)** | **-0.14 (-0.21; -0.08)** | **-0.20 (-0.27; -0.13)** |
|  | PLTd, pictures remembered | Ref | -0.26 (-0.56; 0.04) | -0.12 (-0.41; 0.17) | **-1.03 (-1.33; -0.73)** | **-0.68 (-0.99; -0.37)** | **-0.24 (-0.33; -0.14)** | **-0.27 (-0.37; -0.17)** |
|  | Barthel, index | Ref | -0.01 (-0.09; 0.08) | -0.07 (-0.17; 0.03) | -0.09 (-0.17; 0.01) | -0.06 (-0.16; 0.03) | **-0.05 (-0.08; -0.03)** | **-0.05 (-0.08; -0.02)** |
|  | IADL, points | Ref | -0.01 (-0.14; 0.12) | -0.07 (-0.20; 0.06) | **-0.15 (-0.29; -0.02)** | -0.14 (-0.28; 0.00) | **-0.06 (-0.10; -0.02)** | **-0.11 (-0.15; -0.06)** |

**Abbreviations:** LDCT = Letter-Digit Coding Test; PLTi = Picture-Word Learning test immediate; PLTd = Picture-Word Learning Test delayed; IADL = Instrumental activities of daily living. ^1^=sex, age, country, mean weight during follow-up, height, education. ^2^=sex, age, country, education level, height, mean weight during follow-up time, history of cardiovascular disease, history of diabetes, history of myocardial infarct, smoking, alcohol intake, number of medications, use of diuretics, use of antidepressants. ^3^=sex, age, country, education level, height, mean weight during follow-up time, history of cardiovascular disease, history of diabetes, history of myocardial infarct, smoking, alcohol intake, number of medications, use of diuretics, use of antidepressants, systolic blood pressure variability, mean systolic blood pressure. Items written in bold indicate that the 95% confidence interval does not contain zero.

# **Table S3.** Associations of weight change (slope) during follow-up time and cognitive function at month 30 of follow-up.

|  | | **Weight change (slope)** | | | | | | |
| --- | --- | --- | --- | --- | --- | --- | --- | --- |
|  | | **Low third** | **Low third** | **Middle third** | **Upper third** | **Upper third** | **Continuous^3^** | **Continuous^3^** |
|  | | **Placebo** | **Pravastatin** |  | **Placebo** | **Pravastatin** | **Placebo** | **Pravastatin** |
| **Cognitive test** | | **Beta (95% CI)** | **Beta (95% CI)** | **Beta (95% CI)** | **Beta (95% CI)** | **Beta (95% CI)** | **Beta (95% CI)** | **Beta (95% CI)** |
| **Minimally adjusted^1^** | |  |  |  |  |  |  |  |
|  | Stroop, seconds | **5.72 (3.02; 8.42)** | **3.67 (0.93; 6.41)** | Ref | 0.64 (-2.07; 3.36) | 0.22 (-2.34; 2.78) | **2.27 (1.32; 3.21)** | **1.48 (0.54; 2.41)** |
|  | LDCT, digits coded | **-1.80 (-2.55; -1.04)** | **-1.24 (-2.05; -0.44)** | Ref | -0.20 (-0.96; 0.55) | **0.79 (0.04; 1.55)** | **-0.71 (-0.98; -0.45)** | **-0.67 (-0.94; -0.39)** |
|  | PLTi, pictures remembered | **-0.28 (-0.48; -0.08)** | **-0.49 (-0.69; -0.28)** | Ref | 0.04 (-0.16; 0.24) | -0.04 (-0.23; 0.16) | **-0.14 (-0.20; -0.07)** | **-0.17 (-0.24; -0.10)** |
|  | PLTd, pictures remembered | **-0.51 (-0.79; -0.22)** | **-0.64 (-0.93; -0.34)** | Ref | -0.13 (-0.42; 0.16) | 0.01 (-0.27; 0.29) | **-0.17 (-0.27; -0.08)** | **-0.25 (-0.35; -0.15)** |
|  | Barthel, index | -0.05 (-0.13; 0.03) | -0.03 (-0.13; 0.07) | Ref | -0.03 (-0.12; 0.05) | 0.04 (-0.05; 0.14) | -0.02 (-0.05; 0.00) | -0.02 (-0.05; 0.02) |
|  | IADL, points | -0.07 (-0.20; 0.05) | **-0.16 (-0.29; -0.02)** | Ref | -0.11 (-0.23; 0.02) | -0.03 (-0.15; 0.10) | -0.02 (-0.07; 0.02) | -0.06 (-0.10; 0.00) |
| **Fully adjusted^2^** | |  |  |  |  |  |  |  |
|  | Stroop, seconds | **5.30 (2.51; 8.09)** | **3.37 (0.55; 6.19)** | Ref | 0.49 (-2.32; 3.29) | -0.01 (-2.64; 2.63) | **2.08 (1.11; 3.06)** | **1.46 (0.51; 2.42)** |
|  | LDCT, digits coded | **-1.87 (-2.65; -1.10)** | **-0.95 (-1.77; -0.12)** | Ref | -0.14 (-0.92; 0.63) | **0.95 (0.18; 1.73)** | **-0.74 (-1.01; -0.47)** | **-0.65 (-0.93; -0.37)** |
|  | PLTi, pictures remembered | **-0.29 (-0.50; -0.09)** | **-0.46 (-0.67; -0.24)** | Ref | 0.07 (-0.14; 0.28) | -0.05 (-0.25; 0.16) | **-0.15 (-0.22; -0.08)** | **-0.16 (-0.23; -0.08)** |
|  | PLTd, pictures remembered | **-0.49 (-0.79; -0.20)** | **-0.57 (-0.87; -0.26)** | Ref | -0.09 (-0.39; 0.21) | 0.05 (-0.24; 0.34) | **-0.19 (-0.29; -0.09)** | **-0.25 (-0.35; -0.14)** |
|  | Barthel, index | -0.04 (-0.13; 0.04) | -0.03 (-0.13; 0.08) | Ref | -0.03 (-0.11; 0.06) | 0.03 (-0.07; 0.13) | -0.02 (-0.05; 0.01) | -0.01 (-0.05; 0.02) |
|  | IADL, points | -0.06 (-0.18; 0.07) | **-0.16 (-0.30; -0.02)** | Ref | -0.09 (-0.22; 0.04) | -0.04 (-0.17; 0.09) | -0.02 (-0.07; 0.02) | -0.05 (-0.01; 0.00) |

**Abbreviations:** LDCT = Letter-Digit Coding Test; PLTi = Picture-Word Learning test immediate; PLTd = Picture-Word Learning Test delayed; IADL = Instrumental activities of daily living. ^1^=sex, age, country, mean weight during follow-up, height, education. ^2^=sex, age, country, education level, height, mean weight during follow-up time, history of cardiovascular disease, history of diabetes, history of myocardial infarct, smoking, alcohol intake, number of medications, use of diuretics, use of antidepressants. ^3^ Presented results can be interpreted as the difference is cognitive performance at month 30 per 0.1 kg/month additional weight loss. Items written in bold indicate that the 95% confidence interval does not contain zero.

# **Table S4.** Associations of weight change during follow-up time and cognitive function at month 30 of follow-up.

|  | | **Weight change** |  |  |  |  |
| --- | --- | --- | --- | --- | --- | --- |
|  | | **Weight lost** | **Weight lost** | **Weight stable** | **Weight gained** | **Weight gained** |
|  | | **Placebo** | **Pravastatin** |  | **Placebo** | **Pravastatin** |
| **Cognitive test** | | **Beta (95% CI)** | **Beta (95% CI)** | **Beta (95% CI)** | **Beta (95% CI)** | **Beta (95% CI)** |
| **Minimally adjusted^1^** | |  |  |  |  |  |
|  | Stroop, seconds | **7.16 (4.36; 9.96)** | **5.32 (2.36; 8.28)** | Ref | -1.58 (-4.96; 1.79) | 0.18 (-2.91; 3.27) |
|  | LDCT, digits coded | **-1.27 (-2.06; -0.49)** | **-2.47 (-3.33; -1.61)** | Ref | **1.05 (0.11; 1.99)** | 0.01 (-0.91; 0.92) |
|  | PLTi, pictures remembered | **-0.36 (-0.57; -0.16)** | **-0.61 (-0.84; -0.39)** | Ref | 0.09 (-0.16; 0.34) | -0.04 (-0.27; 0.20) |
|  | PLTd, pictures remembered | **-0.62 (-0.92; -0.32)** | **-0.88 (-1.19; -0.56)** | Ref | -0.11 (-0.46; 0.25) | -0.03 (-0.37; 0.31) |
|  | Barthel, index | -0.03 (-0.11; 0.06) | -0.02 (-0.13; 0.09) | Ref | 0.02 (-0.09; 0.12) | -0.04 (-0.15; 0.08) |
|  | IADL, points | -0.08 (-0.21; 0.05) | -0.10 (-0.25; 0.04) | Ref | -0.04 (-0.20; 0.11) | -0.09 (-0.25; 0.04) |
| **Fully adjusted^2^** | |  |  |  |  |  |
|  | Stroop, seconds | **7.08 (4.20; 9.96)** | **4.30 (1.23; 7.38)** | Ref | -1.57 (-5.04; 1.91) | -0.84 (-4.02; 2.35) |
|  | LDCT, digits coded | **-1.35 (-2.15; -0.54)** | **-2.10 (-2.99; -1.21)** | Ref | **1.20 (0.24; 2.16)** | 0.39 (-0.55; 1.33) |
|  | PLTi, pictures remembered | **-0.40 (-0.61; -0.18)** | **-0.56 (-0.79; -0.33)** | Ref | 0.13 (-0.13; 0.39) | 0.00 (-0.25; 0.24) |
|  | PLTd, pictures remembered | **-0.63 (-0.94; -0.33)** | **-0.78 (-1.11; -0.45)** | Ref | -0.03 (-0.40; 0.34) | 0.07 (-0.28; 0.42) |
|  | Barthel, index | -0.03 (-0.12; 0.06) | 0.00 (-0.12; 0.11) | Ref | 0.04 (-0.07; 0.14) | -0.04 (-0.16; 0.08) |
|  | IADL, points | -0.08 (-0.21; 0.06) | -0.08 (-0.23; 0.07) | Ref | 0.01 (-0.15; 0.16) | -0.07 (-0.23; 0.09) |

**Abbreviations:** LDCT = Letter-Digit Coding Test; PLTi = Picture-Word Learning test immediate; PLTd = Picture-Word Learning Test delayed; IADL = Instrumental activities of daily living. ^1^=sex, age, country, mean weight during follow-up, height, education. ^2^=sex, age, country, education level, height, mean weight during follow-up time, history of cardiovascular disease, history of diabetes, history of myocardial infarct, smoking, alcohol intake, number of medications, use of diuretics, use of antidepressants. Weight loss was defined as ≥5% of body weight decreased from baseline to month 30; weight gain as ≥5% of weight increased; and stability if within <5% weight variation between baseline and month 30 of follow-up. Items written in bold indicate that the 95% confidence interval does not contain zero.

# **Table S5.** Cross-sectional associations of weight variability (SD) and cognitive function at month 30 of follow-up – individuals with disease during follow-up excluded.

|  | | **Weight variability, SD** | | | |
| --- | --- | --- | --- | --- | --- |
|  | | **Low third** | **Middle third** | **Upper third** | **Continuous** |
|  | | **(N=1154)** | **(N=1152)** | **(N=1151)** | **All (N=3457)** |
| **Cognitive test** | | **Beta (95% CI)** | **Beta (95% CI)** | **Beta (95% CI)** | **Beta (95% CI)** |
| **Minimally adjusted^1^** | |  |  |  |  |
|  | Stroop, seconds | Ref | 1.44 (-0.74; 3.63) | **4.64 (2.39; 6.89)** | **1.40 (0.66; 2.13)** |
|  | LDCT, digits coded | Ref | **-1.12 (-1.77; -0.46)** | **-2.09 (-2.77; -1.42)** | **-0.68 (-0.89; -0.46)** |
|  | PLTi, pictures remembered | Ref | -0.14 (-0.30; 0.03) | **-0.57 (-0.75; -0.40)** | **-0.17 (-0.22; -0.11)** |
|  | PLTd, pictures remembered | Ref | -0.09 (-0.33; 0.15) | **-0.84 (-1.09; -0.59)** | **-0.26 (-0.34; -0.18)** |
|  | Barthel, index | Ref | -0.05 (-0.11; 0.02) | **-0.08 (-0.15; -0.01)** | **-0.05 (-0.07; -0.03)** |
|  | IADL, points | Ref | -0.02 (-0.12; 0.09) | **-0.18 (-0.29; -0.07)** | **-0.09 (-0.12; -0.05)** |
| **Fully adjusted^2^** | |  |  |  |  |
|  | Stroop, seconds | Ref | 1.17 (-0.84; 3.19) | **3.98 (1.87; 6.09)** | **1.39 (0.71; 2.07)** |
|  | LDCT, digits coded | Ref | **-1.29 (-1.89; -0.69)** | -2.05 (-2.67; 1.43) | **-0.63 (-0.83; -0.43)** |
|  | PLTi, pictures remembered | Ref | -0.10 (-0.26; 0.05) | **-0.57 (-0.74; -0.41)** | **-0.17 (-0.23; -0.12)** |
|  | PLTd, pictures remembered | Ref | -0.04 (-0.27; 0.18) | **-0.76 (-1.00; -0.52)** | **-0.25 (-0.33; -0.17)** |
|  | Barthel, index | Ref | -0.06 (-0.12; 0.01) | -0.05 (-0.11; 0.02) | **-0.04 (-0.06; -0.02)** |
|  | IADL, points | Ref | -0.05 (-0.13; 0.04) | **-0.11 (-0.20; -0.02)** | **-0.07 (-0.10; -0.04)** |
| **Fully adjusted with systolic blood pressure variability^3^** | | |  |  |  |
|  | Stroop, seconds | Ref | 0.87 (-1.14; 2.88) | **3.31 (1.21; 5.42)** | **1.20 (0.52; 1.87)** |
|  | LDCT, digits coded | Ref | **-1.20 (-1.80; -0.61)** | **-1.85 (-2.47; -1.23)** | **-0.57 (-0.77; -0.37)** |
|  | PLTi, pictures remembered | Ref | -0.09 (-0.24; 0.07) | **-0.53 (-0.70; -0.37)** | **-0.16 (-0.22; -0.11)** |
|  | PLTd, pictures remembered | Ref | -0.02 (-0.25; 0.20) | **-0.72 (-0.95; -0.48)** | **-0.24 (-0.31; -0.16)** |
|  | Barthel, index | Ref | -0.06 (-0.12; 0.01) | -0.04 (-0.11; 0.02) | **-0.04 (-0.06; -0.02)** |
|  | IADL, points | Ref | -0.04 (-0.13; 0.05) | **-0.10 (-0.19; -0.01)** | **-0.07 (-0.10; -0.04)** |

**Abbreviations:** LDCT = Letter-Digit Coding Test; PLTi = Picture-Word Learning test immediate; PLTd = Picture-Word Learning Test delayed; IADL = Instrumental activities of daily living. ^1^=sex, age, country, mean weight during follow-up, height, education. ^2^=sex, age, country, education level, height, mean weight during follow-up time, history of cardiovascular disease, history of diabetes, history of myocardial infarct, smoking, alcohol intake, number of medications, use of diuretics, use of antidepressants. ^3^=sex, age, country, education level, height, mean weight during follow-up time, history of cardiovascular disease, history of diabetes, history of myocardial infarct, smoking, alcohol intake, number of medications, use of diuretics, use of antidepressants, systolic blood pressure variability, mean systolic blood pressure. Stroop test was performed by 2772 participants, the Letter-Digit Coding test was performed by 2857 participants, the immediate Picture-World Learning test was performed by 2889 participants, and the delayed Picture-Word Learning test was performed by 2889 participants. Items written in bold indicate that the 95% confidence interval does not contain zero.

# **Table S6.** Cross-sectional associations of weight change (slope) during follow-up time and cognitive function at month 30 of follow-up - individuals with disease during follow-up excluded.

|  | | **Weight change (slope)** | | | |
| --- | --- | --- | --- | --- | --- |
|  | | **Low third** | **Middle third** | **Upper third** | **Continuous^3^** |
|  | | **(N=1152)** | **(N=1153)** | **(N=1152)** | **All (N=3457)** |
| **Cognitive test** | | **Beta (95% CI)** | **Beta (95% CI)** | **Beta (95% CI)** | **Beta (95% CI)** |
| **Minimally adjusted^1^** | |  |  |  |  |
|  | Stroop, s | **4.21 (2.00; 6.41)** | Ref | -0.12 (-2.29; 2.06) | **1.73 (0.91; 2.56)** |
|  | LDCT, digits coded | **-1.26 (-1.92; -0.60)** | Ref | 0.11 (-0.54; 0.77) | **-0.59 (-0.35; -0.84)** |
|  | PLTi, pictures remembered | **-0.26 (-0.43; -0.09)** | Ref | 0.05 (-0.12; 0.22) | **-0.15 (-0.09; -0.22)** |
|  | PLTd, pictures remembered | **-0.35 (-0.60; -0.11)** | Ref | **0.05 (-0.19; -0.30)** | **-0.18 (-0.09; -0.27)** |
|  | Barthel, index | -0.05 (-0.12; 0.01) | Ref | 0.03 (-0.04; 0.09) | -0.03 (-0.05; 0.00) |
|  | IADL, points | **-0.17 (-0.28; -0.06)** | Ref | -0.06 (-0.17; 0.04) | **-0.06 (-0.10; -0.02)** |
| **Fully adjusted^2^** | |  |  |  |  |
|  | Stroop, s | **4.33 (2.25; 6.41)** | Ref | -0.03 (-2.02; 1.95) | **1.64 (0.90; 2.37)** |
|  | LDCT, digits coded | **-1.30 (-1.92; -0.68)** | Ref | 0.34 (-0.25; 0.94) | **-0.64 (-0.86; -0.43)** |
|  | PLTi, pictures remembered | **-0.37 (-0.53; -0.20)** | Ref | 0.04 (-0.12; 0.19) | **-0.16 (-0.22; -0.11)** |
|  | PLTd, pictures remembered | **-0.45 (-0.69; -0.22)** | Ref | 0.05 (-0.18; 0.27) | **-0.22 (-0.30; -0.13)** |
|  | Barthel, index | -0.03 (-0.09; 0.04) | Ref | 0.01 (-0.06; 0.07) | -0.01 (-0.03; 0.02) |
|  | IADL, points | **-0.10 (-0.19; -0.01)** | Ref | -0.07 (-0.16; 0.02) | -0.03 (-0.06; 0.00) |

**Abbreviations:** LDCT = Letter-Digit Coding Test; PLTi = Picture-Word Learning test immediate; PLTd = Picture-Word Learning Test delayed; IADL = Instrumental activities of daily living. ^1^=sex, age, country, mean weight during follow-up, height, education. ^2^=sex, age, country, education level, height, mean weight during follow-up time, history of cardiovascular disease, history of diabetes, history of myocardial infarct, smoking, alcohol intake, number of medications, use of diuretics, use of antidepressants, systolic blood pressure variability, mean systolic blood pressure. ^3^ Presented results can be interpreted as the difference is cognitive performance at month 30 per 0.1 kg/month additional weight loss. Items written in bold indicate that the 95% confidence interval does not contain zero.

# **Table S7.** Cross-sectional associations of weight change during follow-up time and cognitive function at month 30 of follow-up - individuals with disease during follow-up excluded.

|  | | **Weight change** | | |
| --- | --- | --- | --- | --- |
|  | | **Weight lost** | **Weight stable** | **Weight gained** |
|  | | **(N=558)** | **(N=2477)** | **(N=422)** |
| **Cognitive test** | | **Beta (95% CI)** | **Beta (95% CI)** | **Beta (95% CI)** |
| **Minimally adjusted^1^** | |  |  |  |
|  | Stroop, s | **4.64 (2.20; 7.08)** | Ref | -2.07 (-4.76; 0.61) |
|  | LDCT, digits coded | **-1.61 (-2.33; -0.88)** | Ref | 0.59 (-0.21; 1.39) |
|  | PLTi, pictures remembered | **-0.43 (-0.61; -0.24)** | Ref | 0.09 (-0.12; 0.30) |
|  | PLTd, pictures remembered | **-0.66 (-0.93; -0.39)** | Ref | 0.03 (-0.27; 0.33) |
|  | Barthel, index | -0.05 (-0.12; 0.02) | Ref | 0.02 (-0.07; 0.10) |
|  | IADL, points | **-0.14 (-0.26; -0.02)** | Ref | -0.03 (-0.16; 0.10) |
| **Fully adjusted^2^** | |  |  |  |
|  | Stroop, s | **4.67 (2.42; 6.92)** | Ref | -1.58 (-4.04; 0.89) |
|  | LDCT, digits coded | **-1.64 (-2.30; -0.97)** | Ref | 0.59 (-0.14; 1.32) |
|  | PLTi, pictures remembered | **-0.53 (-0.70; -0.35)** | Ref | 0.01 (-0.19; 0.20) |
|  | PLTd, pictures remembered | **-0.76 (-1.02; -0.51)** | Ref | -0.03 (-0.31; 0.25) |
|  | Barthel, index | -0.04 (-0.12; 0.03) | Ref | -0.03 (-0.11; 0.05) |
|  | IADL, points | **-0.12 (-0.22; -0.02)** | Ref | -0.11 (-0.22; 0.00) |

**Abbreviations:** LDCT = Letter-Digit Coding Test; PLTi = Picture-Word Learning test immediate; PLTd = Picture-Word Learning Test delayed; IADL = Instrumental activities of daily living. ^1^=sex, age, country, mean weight during follow-up, height, education. ^2^=sex, age, country, education level, height, mean weight during follow-up time, history of cardiovascular disease, history of diabetes, history of myocardial infarct, smoking, alcohol intake, number of medications, use of diuretics, use of antidepressants, systolic blood pressure variability, mean systolic blood pressure. Items written in bold indicate that the 95% confidence interval does not contain zero.

# **Table S8.** Continuous weight variability and continuous weight change (slope) in the same model.

|  | | **Weight variable** |  |
| --- | --- | --- | --- |
|  | | **Continuous weight variability (SD)** | **Continuous weight change (slope)^3^** |
| **Cognitive test** | | **Beta (95% CI)** | **Beta (95% CI)** |
| **Minimally adjusted^1^** | |  |  |
|  | Stroop, seconds | **1.71 (1.08; 2.35)** | **1.59 (0.92; 2.26)** |
|  | LDCT, digits coded | **-0.64 (-0.82; -0.46)** | **-0.58 (-0.77; -0.39)** |
|  | PLTi, pictures remembered | **-0.17 (-0.22; -0.12)** | **-0.12 (-0.17; -0.07)** |
|  | PLTd, pictures remembered | **-0.26 (-0.33; -0.19)** | **-0.16 (-0.23; -0.09)** |
|  | Barthel, index | **-0.6 (-0.08; -0.03)** | -0.01 (-0.03; 0.01) |
|  | IADL, points | **-0.09 (-0.12; -0.06)** | -0.02 (-0.05; 0.01) |
| **Fully adjusted^2^** | |  |  |
|  | Stroop, seconds | **1.46 (0.81; 2.11)** | **1.54 (0.85; 2.23)** |
|  | LDCT, digits coded | **-0.58 (-0.77; -0.40)** | **-0.60 (-0.41; -0.80)** |
|  | PLTi, pictures remembered | **-0.16 (-0.21; -0.11)** | **-0.12 (-0.17; -0.07)** |
|  | PLTd, pictures remembered | **-0.24 (-0.31; -0.17)** | **-0.16 (-0.24; -0.09)** |
|  | Barthel, index | **-0.05 (-0.07; -0.03)** | **-0.01 (-0.03; -0.02)** |
|  | IADL, points | **-0.09 (-0.12; -0.05)** | -0.02 (-0.05; 0.01) |

**Abbreviations:** LDCT = Letter-Digit Coding Test; PLTi = Picture-Word Learning test immediate; PLTd = Picture-Word Learning Test delayed; IADL = Instrumental activities of daily living. ^1^=sex, age, country, mean weight during follow-up, height, education. ^2^=sex, age, country, education level, height, mean weight during follow-up time, history of cardiovascular disease, history of diabetes, history of myocardial infarct, smoking, alcohol intake, number of medications, use of diuretics, use of antidepressants.^3^ Presented results can be interpreted as the difference is cognitive performance at month 30 per 0.1 kg/month additional weight loss. Items written in bold indicate that the 95% confidence interval does not contain zero.
